# Hypothesis: may e-cigarette smoking boost the allergic epidemic?

**DOI:** 10.1186/s13601-016-0130-y

**Published:** 2016-11-15

**Authors:** Jean Bousquet, Claus Bachert, Laura Crotty Alexander, Frank T. Leone

**Affiliations:** 1 University Hospital, Montpellier, France; 2 MACVIA-France, Contre les MAladies Chroniques pour un VIeillissement Actif en France, European Innovation Partnership on Active and Healthy Ageing Reference Site, Montpellier, France; 3 INSERM, VIMA: Ageing and Chronic Diseases, Epidemiological and Public Health Approaches, U1168, Paris, France; 4 UVSQ, UMR-S 1168, Université Versailles St-Quentin-en-Yvelines, Versailles, France; 5 University Hospital Ghent, Ghent, Belgium; 6 VA San Diego Healthcare System, San Diego, CA USA; 7 University of California San Diego, San Diego, CA USA; 8 University of Pennsylvania, Philadelphia, PA USA; 9 CHRU Arnaud de Villeneuve, 371 Avenue du Doyen Gaston Giraud, 34295 Montpellier Cedex 5, France

## Background

IgE-associated allergic diseases represent a global health problem increasing in prevalence and severity. An epidemic of IgE-associated allergic diseases has occurred over the past decades globally [1, 2] and many factors driving this epidemic are not clear. The most common diseases (asthma, rhinitis and eczema) are linked, at least partly, to IgE immune response. These diseases are complex multifactorial disorders, with both genetic and environmental components. Reasons explaining the allergy epidemic are not clear. Many inhalants such as air pollution and diesel exhaust particulates are associated with a modulation of the IgE response [3]. On the other hand, tobacco smoking has a minimal effect on the increased prevalence or severity of allergic rhinitis [4].

Any new inhaled compound should be considered a potential adjuvant of the IgE immune response or non-allergic mechanisms leading to a boost in the allergy epidemic. E-cigarettes are largely used to replace conventional cigarette smoking with the intention to reduce known risks for smokers’ health; however, many side effects may still be unknown. Here we focus on the question of whether allergy may be theoretically associated with e-cigarette use.

## E-cigarette vaping

The behaviour of smoking is a cardinal sign of a complex, biosocial compulsive disorder of the brain—a disorder induced by repeated exposure to nicotine [5]. As a result, the global tobacco epidemic claims nearly 6 million lives annually, despite a near-universal appreciation of the catastrophic health consequences of continued use [6]. Ambivalence, or the continuation of maladaptive behaviours in the face of a rational desire to stop, is the hallmark of nicotine dependence, and is frequently resolved by adopting compromise positions, including the use of “light” or filtered cigarettes [7].

The electronic cigarette, or “e-cigarette”, is the latest addition to the list of available compromise products introduced to western markets by the industry. A wide variety of devices are available, with an array of design features and constituent components that significantly influence the pharmacologic/toxicologic profile of each device [8]. Though the devices vary greatly in technical specifications, they most commonly deliver a nicotine-containing aerosol to the aeropharyngeal mucosa, and have been heavily marketed as healthier alternatives to tobacco smoking. In addition to varying amounts of nicotine, the aerosol also delivers propylene glycol and vegetable glycerin, humectants used as a stabilizing vehicle and to create the appearance of smoke plumes, and one or more flavourant additives to increase the appeal of the product [9].

Precisely because the consequences of conventional smoking are so serious, it has been easy for e-cigarette users [10], physicians [11], and at least one professional society [12] to explicitly judge these electronic nicotine delivery devices to be safer than cigarettes. In fact, e-cigarette aerosols do contain far lower concentrations of common cigarette carcinogens on average [13, 14]. However, what is true of the average is not true of the individual products [15]. Neither is it true that carcinogenesis is the sole hazard presented by the aerosol. A growing body of evidence suggests that e-cigarette aerosol constituents may have their own unique hazard profile, distinct from that expected from smoke. For example, a number of disturbances in airway epithelial, endothelial, and inflammatory regulatory physiology have been identified, with uncertain implications on long term health [16, 17].

In high school students in South Korea, e-cigarette users have an increased association with asthma and are more likely to have had days absent from school due to severe asthma symptoms [18].

## Effects of e-cigarettes on human host defence and bacterial virulence

E-cigarette vapor inhaled into human airways interacts with several types of cells, including epithelial and macrophages. Exposure of human cells to e-cigarette vapor has been found to alter innate immune responses and inflammatory signaling [17, 19–22]. In this way, e-cigarette use may induce inflammatory lung diseases or increase susceptibility to invasive bacterial pathogens by effects on mammalian airway cells. Acute e-cigarette vapor inhalation has been found to alter airway physiology: 5 min of e-cigarette use increased airways resistance and lowered the fractional exhalation of nitric oxide (NO), both of which suggest activation of pathways known to be important in asthma [23].

Mammalian cells are not the only residents of human airways. Bacterial pathogens such as *Staphylococcus aureus* (*S. aureus*) commonly colonize the airways and are exposed to inhalants. E-cigarette exposure imposes stress on *S. aureus*, inducing changes in the surface charge, hydrophobicity, and biofilm formation. Nicotine alone had subtle effects, while both aerosolized propylene glycol and vegetable glycerin had dramatic effects on bacterial pathogenicity, independently or when used together. Changes induced by e-cigarette vapor improved the ability of *S. aureus* to adhere to and invade epithelial cells, and increased resistance to human antimicrobial peptides. When unflavored e-cigarette vapor exposed *S. aureus* were introduced into mouse lungs, increased virulence was found via increased bacteria within lung parenchyma and increased mortality [17, 21]. Thus, the use of e-cigarettes may increase the incidence and severity of bacterial lung infections by both direct effects on human cells of host defense as well as effects on bacterial cells. It is unknown whether flavorants will also have pro-virulent effects on bacteria.

## *Staphylococcus* sensitization and allergic diseases

*Staphylococcus aureus* is a frequent colonizer of the upper and lower airways and the skin. In the nose, it may form biofilms and resides intramucosally, and has been associated with different types of T helper cell reactions; recently, arguments for a role also in Th2 immune diseases such as chronic rhinosinusitis and asthma accumulate [24]. *S. aureus* forms a rich immune proteome, with more than 1500 different proteins comprising virulence factors, enterotoxins including classical superantigens, and proteins with enzymatic properties. Whereas the classical enterotoxins may activate T cell populations unspecifically via the variable ß-chain of T cell receptors, other recently discovered molecules such as serine protease-like proteins (spls) obviously elicit a strong Th2-biased immune response and act as allergens or super-allergens, as they induce IgE formation also to inert proteins [25]. Spl-specific memory T cells elaborate Th2 cytokines including IL-4, IL-5 and IL-13, whereas small amounts of IFN-γ, IL-6, TNF and IL-17 are produced. IL-4 and IL-13 drive the immunoglobulin class switch to IgE, and IL-5 orchestrates activation and survival of eosinophils. Both protein families, enterotoxins and spls, have been found in human airway mucosa, and elicit, when given intra-tracheal in mice, an allergic lung inflammation. A typical hallmark of Th2 reactions is the formation of IgE; in the presence of *staphylococcal* superantigens, a polyclonal IgE formation is regularly found including several IgE antibodies directed towards classical and ECG-locus enterotoxins (SE-IgEs); the latter are indicators of a manifest immune reaction to *S. aureus* products. SE-IgE was significantly associated with asthma in 3000 Europeans [26] and was shown to be linked to severe asthma, atopic or non-atopic, both in European [27] and Asian populations [28]. MeDALL (Mechanisms of the Development of Allergy) proposed that *S. aureus* sensitization was associated with a re-occurrence of foetal Type 2 signalling leading to the onset of IgE and non-IgE dependent diseases [29, 30].

## From a hypothesis to public health strategies

The hypothesis that e-cigarette increases *S. aureus* colonisation and then induces sensitization is important to consider since *S. aureus* colonisation is needed for the development of an IgE immune response that is often associated with a polyclonal IgE response, allergic symptoms of the upper and lower airways including allergic rhinitis, severe asthma and/or chronic rhinosinusitis with nasal polyposis.

Although currently there is no confirmation that e-cigarette smoking may induce allergic diseases, there is sufficient background to seriously consider this hypothesis and to test it in appropriate cross-sectional and longitudinal epidemiologic studies. If the effect on *S. aureus* colonisation is important in e-cigarette users, the proof-of-concept should already be demonstrable, as e-cigarettes were introduced to the international market in 2007 and now are used by upwards of 10% of US, European and Asian populations [31]. One possible differentiating feature of e-cigarette-induced allergy would be that users may be prone to develop polysensitization whereas people developing allergic sensitization in adulthood are more often monosensitized or oligosensitized [32].

The next important step will be the identification of e-cigarette components which may induce allergic mechanisms: propylene glycol, glycerine, nicotine, flavorants or toxins produced by the aerosolization process. Animal studies may help guide human studies by narrowing the field of relevant e-cigarette components, defining molecular pathways affected by e-cigarette vapor, and assessing duration and extent of exposure needed to confer increased risk of allergic disease. Large human trials will then be needed to confirm findings with a sufficient power to discriminate between the different types of e-cigarettes.

The most difficult task will be to derive health promotion strategies from this hypothesis. Proponents of e-cigarettes will indicate that allergy and asthma are trivial diseases by comparison to putative cancer prevention [12, 31, 33]. Opponents to e-cigarettes will take the asthma example as a major adverse health consequence of e-cigarette vapor inhalation. In any case, removal of the IgE-promoting components will be needed.

In conclusion, it is urgent to confirm or refute this hypothesis using appropriate studies.

## Abbreviations

IFN: interferon
IL: interleukin
NO: nitric oxide
*S. aureus*: *Staphylococcus aureus*
Spls: serine protease-like proteins
Th2: T helper 2
TNF: tumor necrosis factor
